# Kinetics of Gene Expression Changes in Equine Fetal Interzone and Anlagen Cells Over 14 Days of Induced Chondrogenesis

**DOI:** 10.3389/fvets.2021.722324

**Published:** 2021-08-09

**Authors:** Chan Hee Mok, James N. MacLeod

**Affiliations:** Department of Veterinary Science, Gluck Equine Research Center, University of Kentucky, Lexington, KY, United States

**Keywords:** chondrocyte, development, horse, synovial joint, transforming growth factor beta

## Abstract

Within developing synovial joints, interzone and anlagen cells progress through divergent chondrogenic pathways to generate stable articular cartilage and transient hypertrophic anlagen cartilage, respectively. Understanding the comparative cell biology between interzone and anlagen cells may provide novel insights into emergent cell-based therapies to support articular cartilage regeneration. The aim of this study was to assess the kinetics of gene expression profiles in these skeletal cell lines after inducing chondrogenesis in culture. Interzone and anlagen cells from seven equine fetuses were isolated and grown in a TGF-β1 chondrogenic inductive medium. Total RNA was isolated at ten time points (0, 1.5, 3, 6, 12, 24, 48, 96, 168, and 336 h), and gene expression for 93 targeted gene loci was measured in a microfluidic RT-qPCR system. Differential transcriptional responses were observed as early as 1.5 h after the initiation of chondrogenesis. Genes with functional annotations that include transcription regulation responded to the chondrogenic stimulation earlier (1.5–96 h) than genes involved in signal transduction (1.5–336 h) and the extracellular matrix biology (3–336 h). Between interzone and anlagen cell cultures, expression levels of 73 out of the 93 targeted genes were not initially different at 0 h, but 47 out of the 73 genes became differentially expressed under the chondrogenic stimulation. While interzone and anlagen cells are both chondrogenic, they display clear differences in response to the same TGF-β1 chondrogenic stimulation. This study provides new molecular insight into a timed sequence of the divergent developmental fates of interzone and anlagen cells in culture over 14 days.

## Introduction

During early fetal skeletogenesis, articular cartilage develops from interzone located at the presumptive sites of synovial joints within the cartilaginous anlagen of bones. Therefore, two different types of cartilage differentiate in close proximity. While anlagen cartilage is transient, progressing through endochondral ossification, articular cartilage remains stable and functions throughout life to facilitate biomechanical load distribution and low friction movement between adjoining bones (1, 2). Despite the important functional properties of articular cartilage, its intrinsic ability to restore structural defects is limited in mature mammals (3) in stark contrast to the potential for bone tissue regeneration. Fractured bones repair quite well by recapitulating endochondral ossification; provided the fracture ends are brought together, stabilized, and not compromised by infection or loss of blood supply. Thus, research on the comparative cell biology between interzone and anlagen cells, as well as their developmental chondrogenic pathways may provide novel information relevant to improving mammalian articular cartilage regenerative treatments.

In an earlier study of these two cell types, chondrogenic potential was measured and compared after 21 days in three-dimensional pellet culture and continuous stimulation with a transforming growth factor beta 1 (TGF-β1) chondrogenic induction medium (4). The results demonstrated that while both interzone and anlagen cultures on 21 d showed increased expression of cartilaginous marker genes (aggrecan core protein, ACAN; type II collagen, COL2A1), the cell pellets displayed distinguishing histological characteristics, including proteoglycan amount and distribution, as well as cellular morphology and arrangement. Differences in gene expression and the kinetics of these changes between 0 and 21 d, which presumably would reflect molecular events responsible for differential histological properties of the two cell pellet cultures were not determined. In studies from other groups, the protocol for *in vitro* chondrogenic differentiation has also been reported after 7–28 days, with the expression of extracellular matrix (ECM) genes measured at the mRNA or protein level used as targeted functional outcomes (5, 6). However, gene expression changes induced by TGF-β, a well-established chondrogenic factor, start as early as 30 min−1 h after treatment (7, 8). Therefore, an important knowledge gap is what happens at earlier time points and the kinetics of gene expression differences in response to chondrogenic induction in these two cell types.

By comparing a timed sequence of the cellular response to TGF-β1 induced chondrogenesis between interzone and anlagen cell cultures, the present study investigated how gene expression patterns change over time and molecular details of chondrogenic divergence in these two cell types. The experiments were designed to test the hypothesis that divergent chondrogenic differentiation pathways in interzone and anlagen cultures will be evident within the first 24 h after *in vitro* chondrogenic induction. The aim of the study was to identify critical time points where interesting responses to the chondrogenic stimulation occur in the cell cultures over 14 days.

## Materials and Methods

### Cell Culture and Sample Collection

Primary equine fetal interzone cells, anlagen cells, and dermal fibroblasts (a negative control) were prepared from seven 45-days-old fetuses as previously described [(4); University of Kentucky, Institutional Animal Care and Use Committee approval number, 2014-1215], and stored frozen at passage 2 (P2). For the current experiments, cells were thawed and cultured in high glucose Dulbecco's modified Eagles medium (DMEM; 10569044, Gibco) supplemented with 10% (v/v) fetal bovine serum (heat inactivated; S11150H, Atlanta Biologicals) and 1% (v/v) penicillin/streptomycin (15070063, Gibco). When cell monolayers reached ~80% confluence, they were lifted using 0.25% Trypsin-EDTA solution (25200056, Gibco) and split into new flasks (seeding density of 10,000 cells/cm^2^). Cell viability at passage of >95% was confirmed by the trypan blue dye exclusion test.

When P4 monolayers reached ~80% confluence, one flask was used to collect total RNA. The cells were harvested in a guanidinium thiocyanate solution (1ml/T-75 flask; QIAzol Lysis Reagent; 79306, Qiagen), immediately snap-frozen, and stored at −80°C until total RNA isolation. The additional P4 monolayers were to establish cell pellets at P5 as previously described (4). Each cell pellet (500,000 cells/pellet) was maintained in 1 ml of chondrogenic inductive medium [high glucose DMEM supplemented with 1% penicillin/streptomycin; bovine serum albumin (12.5 mg/ml); ascorbic-2-phosphate (A8960-5G, Sigma, 50 μg/ml); TGF-β1 (human recombinant; T7039-50UG, Sigma-Aldrich, 10 ng/ml); 1% insulin-transferrin-selenium-sodium pyruvate; dexamethasone (100 nM); and 1% non-essential amino acid (11140-050, Gibco)] for the experimental period. The medium was changed every 3 days.

Pellet cultures were collected at ten time points: baseline (0 h), 1.5, 3, 6, 12, 24, 48, 96, 168, and 336 h after the initiation of chondrogenic induction. These time points were selected based on a review of literature relevant to the kinetics of gene expression after TGF-β treatment (7–14). At each time point, the collected pellets were washed with phosphate-buffered saline, snap-frozen, and stored at −80°C until total RNA isolation.

### Total RNA Isolation

Thawed cell monolayer lysates and the pellets (3 pellets/1 ml of the guanidinium thiocyanate solution) were homogenized, then total RNA isolated and purified using spin-columns (RNeasy, Qiagen, 74106) and ethanol precipitation. RNA quantity was determined fluorometrically (Qubit, Q10211, Life Technologies) and purity spectrophotometrically (Nano Drop, Thermo Fisher Scientific). Finally, RNA structural integrity was determined with a Bioanalyzer 2100 (Agilent Technologies) using an Agilent RNA 6000 Pico kit (5067-1513, Agilent Technologies). Monolayer RNA samples resulted in 260/280 ratios of 2.0–2.1, 260/230 ratios of 2.3–2.7, and RINs of 8.7–10, except for one sample with a RIN of 6.8. A substantial majority of cell pellet samples had 260/280 ratios of 1.8–2.1, 260/230 ratios of 1.8–2.7, and RINs of 6.4–10. Out of 210 cell pellet RNA samples, ten samples had 260/230 ratios outside of this range, but gene expression patterns were consistent with experimental group averages, so the data were retained. Any genomic DNA contamination was removed with dsDNase during reverse-transcription using the Maxima First Strand cDNA Synthesis Kit (K1672, Thermo Fisher Scientific).

### Quantitative Polymerase Chain Reaction

#### Targeted Gene Loci

Ninety-three genes of interest (Supplementary Table 1) were selected based on previously generated equine cartilaginous tissue RNA-seq data (15) and a literature review. These genes were either (1) differentially expressed between interzone and anlagen tissue samples at three developmental ages (day-45 gestation, day-60 gestation, and neonatal foals), (2) functionally annotated for fetal development, (3) functionally annotated for chondrogenic differentiation, and/or (4) established components or regulators of TGF-β signaling pathways.

Including three putative endogenous controls, B2M, GUSB, and RPLP0 (16), steady state mRNA levels for a total of 96 gene loci were assessed using commercially available (59 assays) and customized (37 assays) equine-specific TaqMan® primer-probe sets (Thermo Fisher Scientific; Supplementary Table 1). Primer-probe sets designed to span exon junctions (88 assays) were prioritized, with eight assays designed within a single exon.

#### Positive Control RT-qPCR Assessments

Prior to the main microfluidic RT-qPCR analysis, a preliminary assessment was conducted (robotic ViiA™ 7 Real-Time PCR System, Thermo Fisher Scientific) in order to verify (1) amplification of two endogenous controls (GUSB and RPLP0) in all 231 cDNA samples (7 biological replicates × 3 cell lines × 11 time points, including P4 monolayer samples) and (2) amplification of all 96 targeted gene loci by the primer-probe sets in a positive control sample. The positive control was designed to maximize the number of expressed protein-coding genes by pooling equal parts of (1) a pooled total RNA sample composed of 43 different equine tissue/cell sources (17), and (2) a 35-day equine fetus. All 231 samples (10 ng of cDNA/reaction) expressed GUSB [cycle threshold (Ct) 21.44 ± 0.06] and RPLP0 (Ct 18.21 ± 0.06; data not shown). The positive control demonstrated amplification in 95 of the targeted gene loci (Ct 17.68–30.85) with the exception of COL10A1 (data not shown).

#### Microfluidic RT-qPCR

The cDNA samples (10 ng/μl) were arrayed across three 96 well-microfluidic chips (96.96 Fluidigm Dynamic Array). Seven 3-fold dilutions (125, 41.67, 13.89, 4.63, 1.54, 0.51, and 0.17 ng/μl) of the positive control sample were included on each chip to evaluate PCR efficiency and provide an inter-plate control. After 14-cycles of pre-amplification, steady state mRNA levels for the targeted 96 gene loci were measured in a microfluidic RT-qPCR system (Biomark HD high throughput amplification system, Fluidigm) using manufacturer-recommended protocols (18). The data were processed with Fluidigm Real-Time PCR Analysis software.

### Data Analyses and Statistics

**Figure 1:**
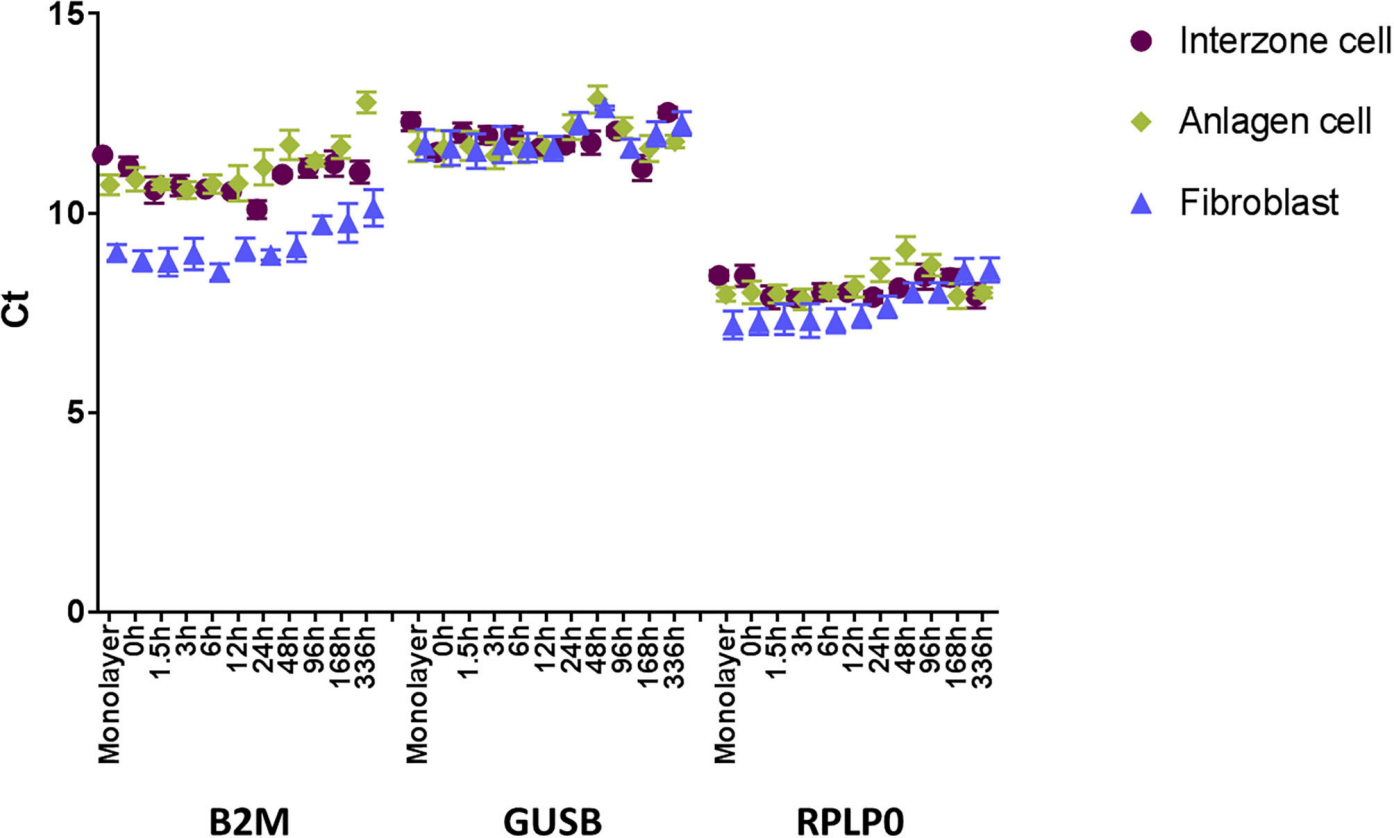
Steady state mRNA levels (Ct, mean ± SEM; *n* = 7) of three prospective endogenous controls (B2M, GUSB, and RPLP0) across the 336-h experimental period measured by microfluidic RT-qPCR. GUSB and RPLP0 displayed more stability across the three cell types and time course.

The two endogenous controls (GUSB and RPLP0) exhibiting the most stable expression across the sample set (Figure 1) were used for normalization within a sample (ΔCt = Ct of a gene of interest – average Ct of GUSB and RPLP0). Then, the ΔCt of each target gene was calibrated with ΔCt of the same target gene in the positive control sample to yield ΔΔCt. Finally, ΔΔCt values were converted to relative quantity [RQ = 2^−ΔΔCt^; fold changes based on expression in the positive control; (19)]. To determine statistical differences between the datum points (targeted gene × cell type × time point), the fold change data were log-transformed and analyzed using SAS statistical software, version 9.3 (SAS Institute Inc.). One-way multivariate analysis of variance was conducted with Tukey's honest significance test for multiple comparison adjustments. The significance threshold was defined as *P* < 0.05.

## Results

Of the 93 targeted genes of interest, six loci were not processed for further data analyses; five genes (ARHGEF15, CHODL, NPY, RET, and STAB1) had minimal expression in experimental samples (RQs <0.02), and one gene (IGF2) had low fluorescent intensity across 5 biological replicates. Microfluidic qPCR data (RQ = 2^−ΔΔCt^ values) for all gene loci investigated are available in Supplementary Table 2. The remaining 87 genes were categorized into three groups based on established functional annotation (Supplementary Table 3): (1) 15 genes regulating transcription, (2) 51 genes involved in signal transduction, and (3) 23 genes involved in ECM biology. Two genes (MASP1 and NEFL) were not categorized into any of the three annotation groups. Four genes were included in two of the three groups; ENTPD1, ENTPD2, and LEF1 in both the transcription and signaling groups, and THBS4 in both the signaling and ECM groups.

### Kinetics of Gene Expression Changes Within Cell Type

Time point differences in steady state mRNA levels for individual gene loci were assessed by comparing values at the given time point to the 0 h baseline within a cell type. Significant upregulation and downregulation events were noted. At the first time point (1.5 h), the significant changes observed were all upregulation. Time points from 3 h on had instances of both upregulation and downregulation. Among the total 261 gene × cell type combinations (87 genes × 3 cell types), 110 combinations showed upregulation (Supplementary Figure 1A), 83 downregulation (Supplementary Figure 1B), and 22 combinations had mixed patterns of upregulation and downregulation (Supplementary Figure 1C). In the other 46 combinations, steady state mRNA levels were stable across all time points (Supplementary Figure 1D).

#### Monolayer vs. 0 h

To assess the effect of trypsin digestion and centrifugation required for establishing cell pellets from monolayers, steady state mRNA levels in P4 cell monolayers were compared to that of P5 0 h cell pellets. Among the 87 targeted loci, only four genes (APLNR, GDF5, S1PR3, and TLR2) had significantly lower expression levels in monolayers compared to 0 h cell pellets in one or more cell types (*P* < 0.05; Supplementary Figure 2). These genes were all in the signal transduction category.

#### Timing of Initial Differential Expression Relative to 0 h Within a Cell Type

**Figure 2:**
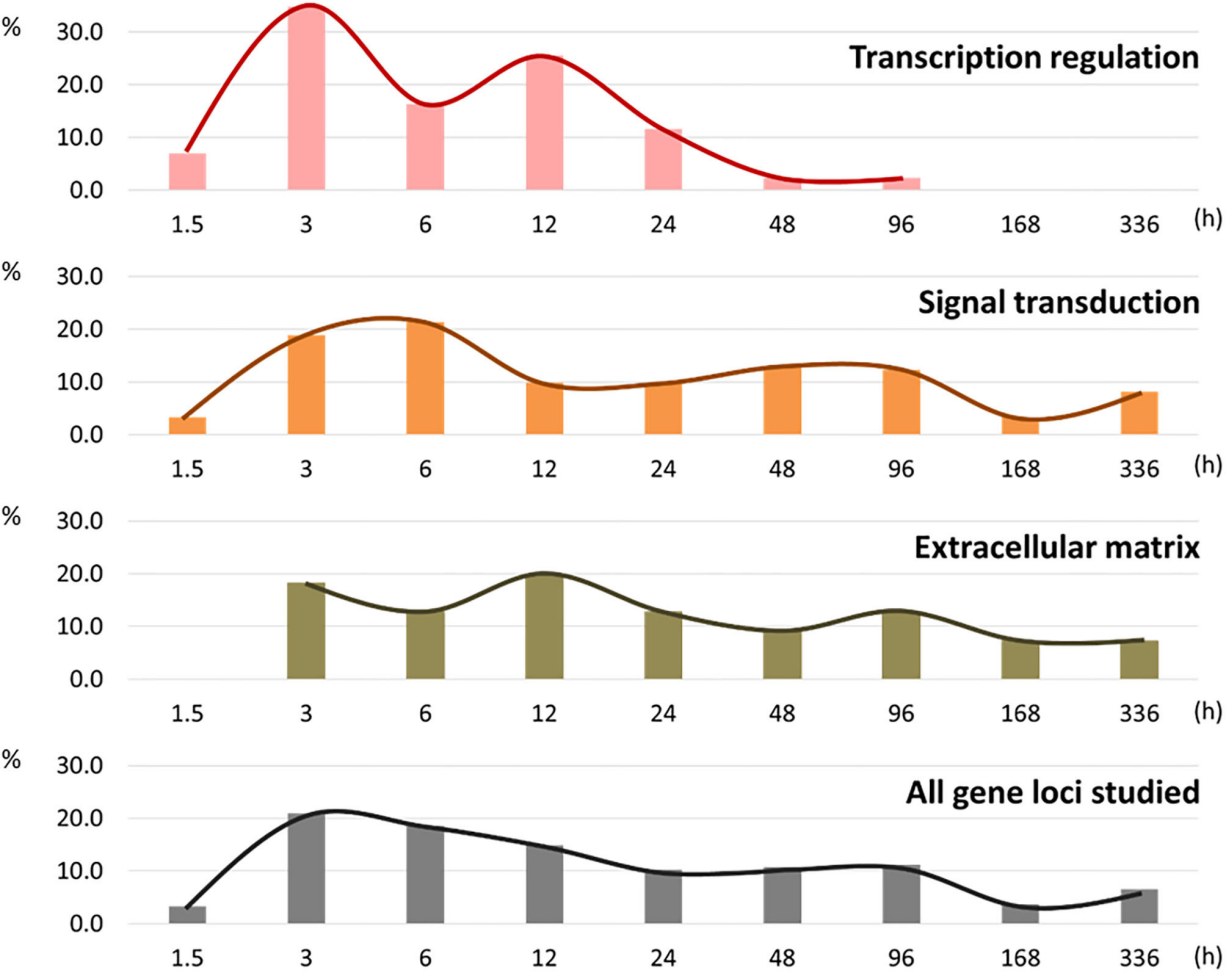
Histogram of percentages of the first response to the chondrogenic stimulation in new gene × cell type combinations within each of the three functional annotation groups.

Eighty-six of the 87 targeted genes displayed at least one significant change in response to chondrogenic induction. The lone exception was GALNT14, which did not change significantly at any time point for the three cell types (Supplementary Figure 1D). Within each functional annotation category, percentages of loci displaying their first onset of change (gene × cell type combinations) were measured (Figure 2; numeric values are available in Supplementary Table 4).

Initial responses to chondrogenic induction were detected at the first collection time point (1.5 h) and involved gene loci with transcription regulation or signal transduction functional annotation. No ECM annotated genes were different at 1.5 h in any cell cultures. The majority of first responses were observed within the first 24 h in all three annotation groups, with peaks at 3 h in the transcription group, 6 h in the signaling group, and 12 h in the ECM group. Most (95.4%) of the initial changes in the transcription regulatory category occurred within the first day (0–24 h), while about 40% of the first responses in the signaling group and the ECM group occurred after 24 h.

In the transcription regulation group, the first half (58.1% of the total) of gene × cell type combinations showed their first chondrogenic responses within 6 h, with the mode observed at 3 h (34.9% of the total). The other 41.9% of gene × cell type combinations first responded to chondrogenic stimulation between 12 and 96 h. Only 4.6% of gene × cell type combinations responded for the first time between 48 and 96 h, and these were observed solely in the negative control, non-chondrogenic fibroblast cultures. In interzone and anlagen cell cultures, steady state mRNA levels for all of the genes with transcription regulation functional annotation had significantly changed within the first 24 h (Table 1).

**Table 1:** Timing of the first response to the chondrogenic induction protocol within functional annotation group in each cell type (% of gene loci with significant changes).

**Annotation group**	**Time point**								
**Transcription regulation**	**1.5 h**	**3 h**	**6 h**	**12 h**	**24 h**	**48 h**	**96 h**	**168 h**	**336 h**
Interzone cell	6.7	33.3	20.0	26.7	13.3	0.0	0.0	0.0	0.0
Anlagen cell	7.1	28.6	14.3	28.6	21.4	0.0	0.0	0.0	0.0
Fibroblast	7.1	42.9	14.3	21.4	0.0	7.1	7.1	0.0	0.0
**Signal transduction**									
Interzone cell	0.0	27.8	19.4	5.6	13.9	11.1	11.1	2.8	8.3
Anlagen cell	4.3	17.0	25.5	14.9	6.4	17.0	10.6	2.1	2.1
Fibroblast	5.1	12.8	17.9	7.7	10.3	10.3	15.4	5.1	15.4
**Extracellular matrix**									
Interzone cell	0.0	23.5	5.9	23.5	17.6	11.8	5.9	11.8	0.0
Anlagen cell	0.0	19.0	14.3	28.6	4.8	9.5	9.5	4.8	9.5
Fibroblast	0.0	11.8	17.6	5.9	17.6	5.9	23.5	5.9	11.8

*Progressively darker colors are used to illustrate higher percentages of gene loci. Transitions between colors occur at 5% intervals (0–5%; 5.1–10%; 10.1–15%; 15.1–20%; 20.1–25%; 25.1–30%; 30.1–35%; 35.1–40% (No data point); 40.1–45%)*.

In comparison to the transcription regulation group, genes involved in signal transduction showed slightly delayed responses. Roughly half (53.3%) of the gene × cell type combinations changed significantly within the first 12 h. Response to chondrogenic induction was even more delayed for genes involved in ECM biology. Indeed, no differences were observed in this annotation group at 1.5 h. The first half (50.9%) of gene × cell type combinations were observed between 3 and 12 h (Figure 2).

In the negative control dermal fibroblasts, responses to chondrogenic induction were delayed compared to interzone and anlagen cells. Transcriptional regulatory genes all showed their first responses within 24 h in the chondrogenic cells, compared to some initial changes delayed until 96 h in fibroblast cultures. In the signal transduction group, 8.3 and 2.1% of the first responses were recorded at the last time point (336 h) in interzone and anlagen cell cultures, respectively, while 15.4% of those was recorded at 336 h in fibroblast cultures. Also, the majority of first reactions in the ECM group were at 3 h (23.5%) and 12 h (23.5%) in interzone cultures, 12 h (28.6%) in anlagen cultures, and 96 h (23.5%) in fibroblast cultures (Table 1).

### Relative Differences Between Cell Types at Each Time Point

Steady state mRNA levels at each time point were compared in pairwise comparisons between cell types, and the results categorized into four groups (Table 2; Supplementary Figure 3). While 14–18 genes were already differentially expressed at 0 h in the comparisons between cell types, most of the targeted loci (69–73 of the 87) did not show differences initially.

**Table 2:** Four patterns of differential gene expression before and after inducing *in vitro* chondrogenesis.

**Cell type comparison**	**Already different at baseline (0 h)**		**Not different at baseline (0 h)**	
	**Retained differences after chondrogenesis**	**Lost differences after chondrogenesis**	**Became different after chondrogenesis**	**Remained similar after chondrogenesis**
IZ vs. ANL	11 genes	3 genes	47 genes	26 genes
IZ vs. FB	14 genes	0 gene	41 genes	32 genes
ANL vs. FB	18 genes	0 gene	47 genes	22 genes
	3 genes:	0 gene	15 genes:	7 genes:
Common genes in all comparisons	DLX5, LEF1, and RUNX3		ADAMTS5, ANGPTL4, COL5A3, ENTPD2, FGF18, FGFR3, IHH, RELN, S100A4, SERPINE1, SGMS2, SLC38A1, SNAI1, TLR2, and WNT9A	ADGRG2, BMP2, COL10A1, KCNJ8, LOC100630171, PTCH2, and SPARCL1
	5 genes:	3 genes:	9 genes:	12 genes:
Specific to the comparison between IZ and ANL cultures	GDF6, MGP, OMD, PDLIM1, and RUNX2	COL2A1, COMP, and DIO2	ALPK3, ASS1, AQP1, CTGF, FAM20A, FGF1, ITGA7, PLVAP, and TLR4	ADGRG1, ALPL, APLNR, DCN, ENTPD1, FAM132A, FZD1, GDF5, IGFBP5, IGFBP7, MMP2, and PLAT

*IZ, interzone cell; ANL, anlagen cell; FB, fibroblast*.

#### Genes With Differential Expression Levels Prior to the Chondrogenic Induction

Across all three cell type comparisons, there were three common genes (DLX5, LEF1, and RUNX3) that had different baseline expression levels at 0 h, while also displaying cell type-specific responses to chondrogenic induction. Specific to comparisons between interzone and anlagen cultures, five genes (GDF6, MGP, OMD, PDLIM1, and RUNX2) started with different expression levels and also retained differential profiles following the chondrogenic induction (Table 2; Supplementary Figure 3A).

If initial mRNA levels of a gene were different in the comparisons to fibroblast cultures, the gene also showed different expression levels after inducing chondrogenesis. However, between interzone and anlagen cell cultures, three genes (COL2A1, COMP, and DIO2) with different initial mRNA levels lost differences across all post-chondrogenic induction time points (Table 2; Supplementary Figure 3B).

#### Genes With No Differential Expression Levels Prior to the Chondrogenic Stimulation

Among the 69–73 genes that initially did not have different expression levels in pairwise cell-type comparisons, 41–47 genes developed significant differences in steady state mRNA levels after inducing chondrogenesis. Between interzone and anlagen cultures, there were 47 genes in this category, but only nine (ALPK3, ASS1, AQP1, CTGF, FAM20A, FGF1, ITGA7, PLVAP, and TLR4) were specific to the comparison between these two chondrogenic cell lines (Table 2; Supplementary Figure 3C).

On the other hand, seven genes showed no difference in any cell type comparison: ADGRG2, BMP2, COL10A1, KCNJ8, LOC100630171, PTCH2, and SPARCL1. Focusing only on interzone and anlagen cultures, however, twelve additional genes responded to chondrogenic induction without cell-type differences: ADGRG1, ALPL, APLNR, DCN, ENTPD1, FAM132A, FZD1, GDF5, IGFBP5, IGFBP7, MMP2, and PLAT (Table 2; Supplementary Figure 3D).

#### Individual Gene Loci Demonstrate Clear Examples of Cell Type-Specific Differences

**Figure 3:**
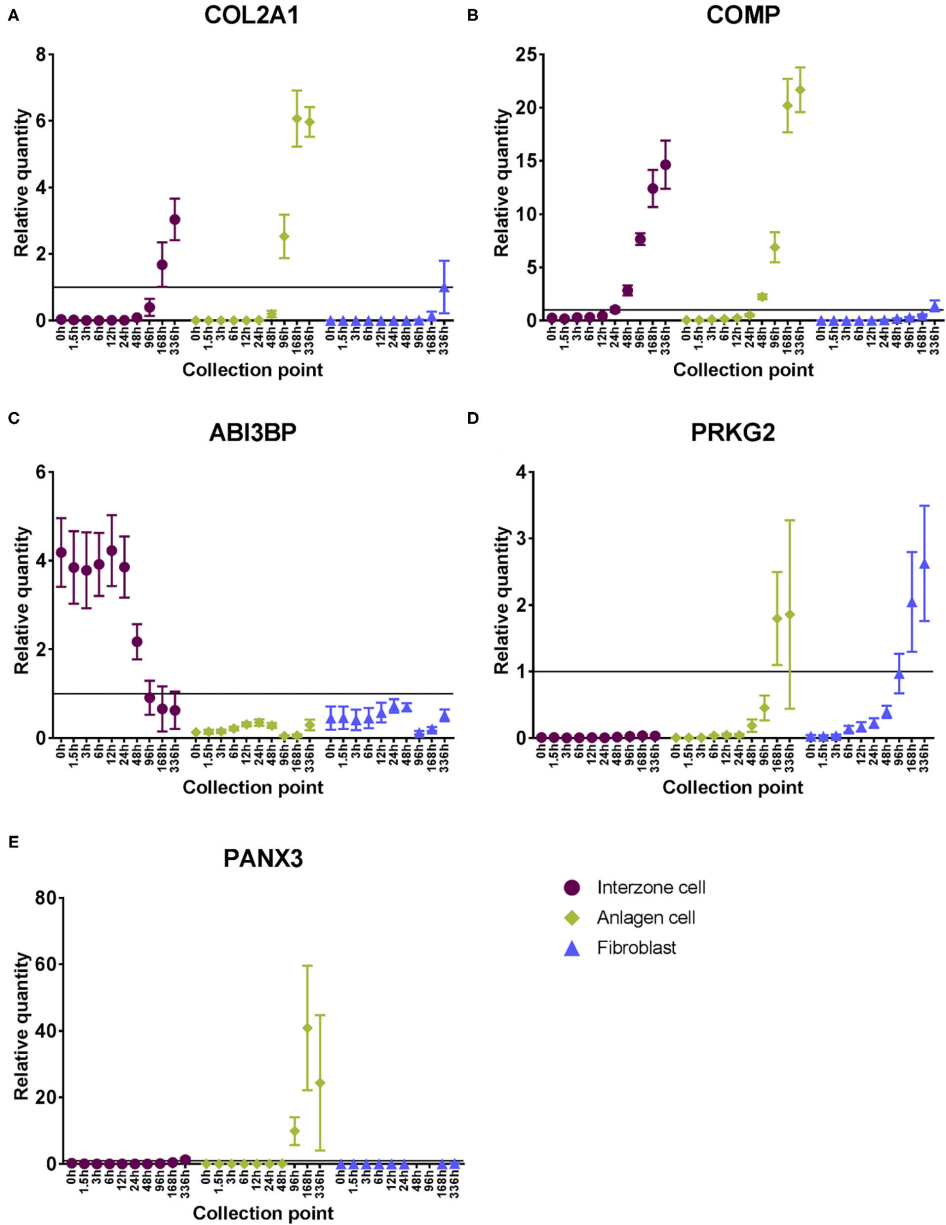
Steady state mRNA levels of example cell type-specific expression profiles across the experimental period. Relative quantities were calculated based on the positive control sample (pooled equine adult tissue and fetus RNA). Mean ± SEM (*n* = 7); Classic cartilaginous biomarkers: **(A)** COL2A1 and **(B)** COMP; Example genes that were differentially regulated in interzone cell cultures compared to the other cell types: **(C)** ABI3BP was downregulated and **(D)** PRKG2 was minimally expressed in interzone cell cultures; Example gene that was differentially regulated in anlagen cell cultures compared to the other cell types: **(E)** PANX3 was upregulated only in anlagen cell cultures toward later time points.

Cartilage biomarkers (COL2A1 and COMP) were upregulated in the chondrogenic cell lines over time (Figures 3A,B). While their steady state mRNA levels were also increased in fibroblast cultures toward the end of the experimental period, the levels were consistently lower compared to the chondrogenic cell cultures (Supplementary Figures 3A,C).

There were examples of genes that displayed unique expression patterns in interzone or anlagen cultures compared to the other two cell types. For interzone cells, changes in steady state mRNA levels of ABI3BP showed a clear example of downregulation (Figure 3C), while PRKG2 is an example of minimal expression (Figure 3D). In contrast, PANX3 was upregulated only in anlagen cell cultures toward the later time points, while its mRNA levels were consistently lower or not detected in the other two cell cultures (Figure 3E).

## Discussion

### Relationship Between Early and Delayed Gene Expression Changes

The results from the present study demonstrate a sequence whereby genes encoding proteins functionally annotated in transcription, signaling events, and ECM are expressed in roughly that order. The model consistent with this observation is that transcription regulating genes are primary responders, followed by secondary changes in downstream signaling events alter effector genes involved in ECM biology. To further elucidate these relationships and define “(early) primary” and “(delayed) secondary” genes from a perspective of cell biology, an experiment designed to include protein synthesis inhibitors will be necessary (20).

It is interesting to note that all changes in mRNA levels observed at 1.5 h were upregulation compared to the baseline. On the other hand, downregulation started to be detected by 3 h. This may confirm that degradation of transcripts requires more time than *de novo* synthesis (21). Regulatory genes may have relatively faster decay rates in order to rapidly alter signal transduction; 16 out of the 18 genes that showed their first responses as downregulation between 3 and 6 h were involved in either transcription or signaling events.

While the present study suggests some interesting time points where major regulatory responses to the chondrogenic stimulation likely occur, additional data on a transcriptome level will be needed to reveal important regulatory mechanisms responsible for the divergent chondrogenic pathways between interzone and anlagen cells. This knowledge would provide new information relevant to therapeutic efforts to improve articular cartilage regeneration.

### Differential Gene Regulation Between Cell Types

#### Differences Between Chondrogenic and Non-chondrogenic Cell Cultures

Consistent with their established functional annotations, COL2A1 and COMP, classic cartilaginous biomarker genes (22, 23), were significantly upregulated in interzone and anlagen cell cultures confirming their chondrogenic potential. Overall, delayed responses in all functional annotation categories were observed in the fibroblasts compared to the chondrogenic cell lines. While this is likely due to the chondrogenic potential of interzone and anlagen cells, it is important to note that the targeted genes were selected based in part on established annotations related to chondrogenesis and expression in skeletal tissues. A full transcriptome analysis would help to determine if the relationship to chondrogenesis is a key variable.

#### Differences Between Interzone and Anlagen Cell Cultures

Interzone and anlagen tissues in equine fetuses at day-45 of gestation are readily distinguished morphologically with a dissecting microscope. Not surprisingly some gene expression differences were noted, with 14 out of the 87 targeted gene loci starting at different mRNA levels. Among the 14 genes, 11 genes still expressed different profiles after inducing chondrogenesis, and five of them showed these differential patterns only between interzone and anlagen cultures, but not in the comparisons to dermal fibroblasts. OMD and RUNX2 were upregulated in anlagen cell cultures after the chondrogenic induction, and their known annotation is related to ECM production in the bone (24, 25). Thus, the results are consistent with anlagen cells having developmental pathways leading to bone formation. Type X collagen (COL10A1) is a well-established biomarker of hypertrophic chondrocytes (26, 27). This was the single gene locus from the panel that was not detected in the positive control sample, a finding explained by the fact that the pool of RNAs used did not include hypertrophic cartilage. However, with the transcript pre-amplification steps used in the Fluidigm protocol, RQ values for COL10A1 could be calculated but did not exhibit significant cell type differences (Supplementary Figure 4). Subsequent RNA-seq analyses on five of the earlier time points (0, 1.5, 3, 12, and 96 h) confirmed no cell type differences (data not shown).

On the other hand, MGP and GDF6 were upregulated in interzone cultures, and their reported functional annotations are inhibiting ectopic tissue calcification (28) and diarthrodial joint formation (29), respectively. In addition, mRNA levels of PDLIM1 were greater in interzone cell cultures at the latest time points. From a previous RNA-seq dataset (Adam et al., in preparation), this gene was not differentially expressed between interzone and anlagen tissue lineages from 45-day-old equine fetuses and neonatal foals, but its expression was 3.41-fold greater (*P* < 0.0001) in interzone tissue compared to cartilaginous anlagen from 60-day-old fetuses. These observations may suggest that this transcription regulatory gene might become upregulated in interzone at a later stage during articular cartilage development. Taken together, expression patterns of these genes in the culture model are consistent with the articular and hypertrophic cartilaginous tissue outcomes of interzone and anlagen cells, respectively.

As noted above, steady state mRNA levels for 73 of the 87 targeted gene loci were not significantly different at 0 h, and the expression of these genes either diverged or changed in similar ways during the time course. Forty-seven genes differentially responded to the chondrogenic induction, but only nine of these were specific to the comparison between interzone and anlagen pellets. FGF1, a gene expressed in proliferative and hypertrophic chondrocytes (30), was upregulated early (6 h) in anlagen cultures and justifies further investigation. For the other 26 genes, the changes were concordant after chondrogenic induction. GDF5 is an established interzone biomarker (2, 31), but in the current *in vitro* model using primary cells derived from 45-day-old equine fetuses, its mRNA profiles were not different between interzone and anlagen cultures at both pre- and post-chondrogenic induction time points. The discrepancy may be due to the important variable of fetal age (developmental stage) between the two sample sets, but cell signaling and species-specific parameters cannot be excluded. Similarly, while ALPL is known to be involved in bone mineralization (32), its mRNA levels were not different between the two chondrogenic cell cultures. This may not be surprising given that the culture system was not intended to model osteogenesis.

#### Cell Type-Specific Expression Profiles

Several gene loci displayed clear cell type-specific expression profiles. Examples in interzone cell cultures were ABI3BP and PRKG2. ABI3BP is a novel ECM gene and is required to switch the cellular status from proliferation to differentiation in mesenchymal stem cells, including during chondrogenesis (33). The mRNA levels of this gene were initially greater in interzone cell cultures, started to decrease by 96 h, and became as low as the other cell lines at the last time point. In one study, this gene was found to be differentially upregulated in articular cartilage compared to hypertrophic growth plate both *in vivo* and *in vitro* (34). In contrast, mRNA levels of PRKG2 were minimal and remained low in interzone cultures, but were upregulated in the other two cell lines at later time points. PRKG2 is known to be involved in mammalian skeletal development, and its null mutation resulted in 23–30% decreased length of limb bones (35) due to impaired chondrocyte hypertrophy (36). Finally, PANX3 was specifically upregulated in anlagen cell cultures. This gene is expressed in cartilaginous anlagen of a developing limb (37), and when PANX3 was knocked down, hypertrophic differentiation was delayed and attenuated (38).

In conclusion, the data supported the hypothesis that divergent chondrogenic pathways in interzone and anlagen cell cultures will be evident within an early time frame—within the first 24 h after inducing *in vitro* chondrogenesis. The results confirmed the chondrogenic potential of interzone and anlagen cells, but also documented distinct functional responses to the same TGF-β1 chondrogenic induction signal. Several gene expression profiles were broadly consistent with well-established developmental fates of interzone and anlagen cells within limb buds. Overall, transcription regulatory responses preceded changes in genes with functional annotation related to signal transduction or ECM biology. Further investigation at a transcriptome level, together with functional experiments, should help identify key regulators and gene ontology relationships relevant to the mechanisms underlying divergent chondrogenesis between interzone and anlagen cells.

## Data Availability Statement

The original contributions presented in the study are included in the article/Supplementary Material, further inquiries can be directed to the corresponding author/s.

## Author Contributions

JM: secured funding. CM and JM: study design, data analysis, and drafting and revision. CM: data acquisition. All authors contributed to the article and approved the submitted version. All authors contributed to the article and approved the submitted version.

## Conflict of Interest

The authors declare that the research was conducted in the absence of any commercial or financial relationships that could be construed as a potential conflict of interest.

## Publisher's Note

All claims expressed in this article are solely those of the authors and do not necessarily represent those of their affiliated organizations, or those of the publisher, the editors and the reviewers. Any product that may be evaluated in this article, or claim that may be made by its manufacturer, is not guaranteed or endorsed by the publisher.
