# Supplementary material for: Kinetics of Gene Expression Changes in Equine Fetal Interzone and Anlagen Cells Over 14 Days of Induced Chondrogenesis
Source: Front Vet Sci. 2021 Aug 9;8:722324. doi: 10.3389/fvets.2021.722324 (PMC8380811; doi:10.3389/fvets.2021.722324)
Supplement: Supplementary file 5 [file Data_Sheet_1.pdf]

a) Upregulated genes

(Time point, h)

Genes	Cell type	1.5	3	6	12	24	48	96	168	336
SNAI1	FB	***	***	***	***	***	***			
IHH	ANL	***	***	***	***	***	***	***	***	***
IHH	FB	*	**			**	***	***	***	***
S1PR3	ANL	**	***	***	**	***	***	***	***	***
S1PR3	FB	**	***	***	***	***	***	***	***	***
ALPK3	IZ		***	***						
RUNX3	IZ		***	**						
RUNX3	ANL		***							
RUNX3	FB		***	***						
ANGPTL4	IZ		***	***	***	***	***	*		
ANGPTL4	FB		***	***	***	***	***	***	***	***
AQP1	IZ		*	**	**	***	***	***	***	***
AQP1	ANL		***	***	***	***	***	***	***	**
AQP1	FB		**	***	***	***	***	***	***	***
FAM132A	IZ		*	**	***	***	***	***	***	***
FGF1	ANL		**	***	***	***	***	***	***	***
IHH	IZ		*		***	***	***	***	***	***
S1PR3	IZ		***	**	*	***	***	***	***	***
WNT9A	IZ		**	***						
WNT9A	FB		*	**	***	**	*	***	***	***
COL5A3	IZ		***	***	***	***	***	***		
COL5A3	ANL		***	***	***	***	***	**		
COL5A3	FB		***	***	***	***	***	***	***	
COMP	ANL		*	***	***	***	***	***	***	***
SGMS2	IZ		***							
SGMS2	ANL		*						**	*
THBS4	IZ		*	***	***	***	***	***	***	***
THBS4	ANL		***	***	***	***	***	***	***	***
THBS4	FB		**	***	***	***	***	**	***	***
ASS1	FB			***	***	***	***	*		*
LEF1	IZ			**		*	***	**	***	**
BMPR1A	ANL			**	***					
BOC	ANL			**	**		**		**	***
CDON	IZ			*	*	***				
CDON	ANL			***	***	**	**		***	***
FAM132A	ANL			*	***	***	***	*	***	**
GDF5	ANL			**	***	***	***	***	***	***
KCNJ8	ANL			***	***		***	***		
LOC100630171	ANL			*						
LOC100630171	FB			**					**	
PRKG2	FB			***	***	***	***	***	***	***
ALPL	FB			***	***	***	***	**	**	***
OMD	ANL			***	***	***	***	***	***	***
OMD	FB			**	***	***	***	***	***	***
RELN	ANL			*	*	***	***	***	***	***
TGFBI	IZ			***	***	***	***	***	***	
ASS1	IZ				***	***	***		*	**
ASS1	ANL				***	***	***			
ENTPD2	ANL				*	**	***	*	***	***
ENTPD2	FB				***	***	***			
RUNX2	IZ				**	***	***	***		
RUNX2	ANL				**	***	***	**		
RUNX2	FB				***	***	***	***	***	**
BOC	FB				*	**	***	***	***	*
GDF5	IZ				***	***	***	***	***	***
ALPL	IZ				*	*				
ALPL	ANL				**	**	*			
DCN	IZ				***	***	***	***	***	***
DCN	ANL				*	***	***	***	***	***
FAM20A	ANL				**	***	***	***	*	
MMP2	ANL				*	***	***	***	***	**
OMD	IZ				***	***	***	***	***	***
SMPD3	ANL				***	***	***	***	***	***

(continued Supplementary Figure 1.a)

(Time point, h)

Genes	Cell type	1.5	3	6	12	24	48	96	168	336
ENTPD2	IZ					**	**			
LEF1	ANL					*	***	***		
SP7	IZ					**	**	***	**	**
SP7	ANL					**	***	***	***	
BOC	IZ					**				
S100A1	IZ					***	***	***	***	***
S100A1	ANL					***	***	***	***	***
S100A1	FB					**	***	***	***	***
TNFSF11	FB					***	***	***	***	***
COL2A1	ANL					*	***	***	***	***
COMP	IZ					***	***	***	***	***
COMP	FB					***	***	***	***	***
MMP2	IZ					***	***	***	**	*
MMP2	FB					***	***	***	***	***
SPARCL1	IZ					**	***	***	***	***
SPARCL1	FB					***	***	***	***	***
ABCC9	ANL						**	**		
BMP2	ANL						**	***	**	***
FGF1	IZ						**		*	
FGF1	FB						***			
ITGAV	FB						***	**		
PLVAP	FB						***	***	**	
PRKG2	ANL						***	***	***	***
TNFSF11	ANL						***	***	**	
MGP	ANL						*	***	***	***
RELN	IZ						***	***	***	***
SPARCL1	ANL						*	***	***	***
APLNR	FB							**	***	***
BMP2	IZ							*		
FGFR3	IZ							***	***	***
FGFR3	ANL							***	***	***
FRZB	ANL							***	***	
GDF5	FB							**	***	***
IBSP	FB							**	*	
PANX3	ANL							**	***	**
PLVAP	IZ							**	***	**
DCN	FB							*	**	**
FAM20A	FB							*	*	
SMPD3	FB							*	***	***
TLR2	FB								***	***
COL10A1	ANL								**	*
FAM20A	IZ								***	*
RELN	FB								**	***
SMPD3	IZ								**	***
BMP2	FB									**
NTRK2	FB									**
COL2A1	FB									***

Annoation group
Trascnription regulation
Signal transduction
Extracellular matrix
Not categorized

Upregulated based on 0h	***, P<0.0001
	**, P<0.01
	*, P<0.05

b) Downregulated genes

(Time point, h)

Genes	Cell type	1.5	3	6	12	24	48	96	168	336
OSR2	IZ		*				*			
OSR2	ANL		***	***	*	***	***			
OSR2	FB		***	**		**	**		**	***
SNAI2	FB		***	***						
CTGF	ANL		***	***	***	***	***	**	**	***
CTGF	IZ		***	***	***	***	***			
CTGF	FB		**	***	***	***	***	***	**	**
DIO2	ANL		**	*	***					
GLI3	IZ			***	*					*
GLI3	ANL			***		*				***
GLI3	FB			***		**	*		**	***
CREB5	IZ			**	**			***	***	***
CREB5	ANL			**				***	***	***
ADGRG2	IZ			**	***	***	***	*	***	
GDF6	IZ			***			*			*
GDF6	ANL			***	**	***	**	**	***	***
GDF6	FB			***	**	***	***	*		*
MET	IZ			**	***	***	***	***	***	***
MET	ANL			**	***	***	***	***	***	***
MET	FB			***	***	***	***	***	***	***
PTCH2	IZ			***	***	***	***	***	*	
PTCH2	ANL			***	***	***	***	***		**
PTCH2	FB			***	***	***	***	***	***	***
SLC38A1	IZ			*	***	***	***	***	***	***
SLC38A1	ANL			*	***	***	*	***		
SLC38A1	FB			***	***	***	***	***	***	***
TLR4	FB			**	**					***
TNFRSF21	ANL			*	***	***	***		***	***
ADAMTS5	FB			*	*					
PDLIM1	IZ				***	***	***	***	***	***
ENTPD1	IZ				***	***	***	**	**	***
ENTPD1	ANL				**	***	***	***	***	***
ENTPD1	FB				**	***	***	***	***	***
ADGRG2	ANL				**	***	***	**		
TLR4	ANL				*	**			***	***
SHC3	ANL				*	**			***	**
FGF18	ANL				**			**	***	***
ADAMTS5	IZ				***	***	***	***	***	***
ADAMTS5	ANL				**	***	**	***	***	***
MASP1	IZ				**	***	***	***	***	***
MASP1	ANL				**	***	***	***	***	***
MASP1	FB				**	***	***	***	***	***
PDLIM1	ANL					***	***	***	***	***
IGFBP7	FB					*	***	***	***	***
SERPINE1	IZ					**	***	***	***	***
TLR4	IZ					**				
TSPAN15	ANL					**	***	***	***	***

(continued Supplementary Figure 1.b)

(Time point, h)

Genes	Cell type	1.5	3	6	12	24	48	96	168	336
PDLIM1	FB						***	***	***	***
FRZB	FB						***	**	***	**
IGFBP7	IZ						**	***	**	
IGFBP7	ANL						***	*		
PLAT	IZ						*	***	***	***
SERPINE1	ANL						**	***	***	***
TSPAN15	IZ						***	***	***	***
ITGA7	IZ						**	***	**	***
ITGA7	FB						**	***	***	***
NEFL	IZ						***	***	***	***
CREB5	FB							***	***	***
ADGRG1	FB							***	***	***
CDH13	ANL							***	***	***
CDH13	FB							***	***	***
IGFBP5	IZ							**	***	***
PLAT	ANL							***	***	***
SERPINE1	FB							***	***	***
ABI3BP	IZ							**	***	***
ITGA7	ANL							*	**	***
TIMP2	ANL							**	***	***
TIMP2	FB							**		*
NEFL	ANL							***	**	
CDH13	IZ								***	***
IGFBP5	FB								**	***
S100A4	ANL								***	***
FZD1	IZ									***
FZD1	ANL									***
ITGAV	IZ									*
PLAT	FB									**
S100A4	IZ									***
S100A4	FB									**
TNFRSF21	FB									**
TSPAN15	FB									**
CLU	ANL									***
CLU	FB									***
COL1A1	ANL									***

Annoation group
Trascnription regulation
Signal transduction
Extracellular matrix
Not categorized

Downregulated based on 0h	***, P<0.0001
	**, P<0.01
	*, P<0.05

c) Genes with mixed upregulation and downregulation patterns

(Time point, h)

Genes	Cell type	1.5	3	6	12	24	48	96	168	336
SNAI1	IZ	***	***	***	***	***	**			**
SNAI1	ANL	***	***	***	***	***	***			*
ALPK3	ANL		***	***		***	***			***
ALPK3	FB		***	***		**	*		**	**
SNAI2	IZ		**	**			*	**		
SNAI2	ANL		***	***				***	***	***
DLX5	IZ		*				*	***	***	**
DLX5	FB		**					***	***	***
SP7	FB		**				*	**		*
ADGRG1	IZ		**	**			*	***	***	***
ADGRG1	ANL		**	**				***	***	***
ANGPTL4	ANL		***	***	***	***	***	***		***
WNT9A	ANL		**	***	*				**	***
TNFSF11	IZ		**				*	***	***	***
COL2A1	IZ		*	**					***	***
TGFBI	ANL			***	***	***	***	***		*
NEFL	FB			**	***			***	***	***
IGFBP5	ANL				**	***	***			***
TGFBI	FB				***	***	***			***
FAM132A	FB					*	**			***
PLVAP	ANL						**	*		*
ITGAV	ANL						**			***

Annoation group
Trasncription regulation
Signal transduction
Extracellular matrix
Not categorized

Upregulated based on 0h	***, P<0.0001
Downregulated based on 0h	**, P<0.01
	*, P<0.05

d) Genes with no response to the chondrogenic stimulation

(Time point, h)

Genes	Cell type	1.5	3	6	12	24	48	96	168	336
DLX5	ANL									
LEF1	FB									
ABCC9	IZ									
ABCC9	FB									
ADGRG2	FB									
APLNR	IZ									
APLNR	ANL									
BMPR1A	IZ									
BMPR1A	FB									
CDON	FB									
DIO2	IZ									
DIO2	FB									
FGF18	IZ									
FGF18	FB									
FGFR3	FB									
FRZB	IZ									
FZD1	FB									
IBSP	IZ									
IBSP	ANL									
KCNJ8	IZ									
KCNJ8	FB									
LOC100630171	IZ									
NTRK2	IZ									
NTRK2	ANL									
PANX3	IZ									
PANX3	FB									
PRKG2	IZ									
SHC3	IZ									
SHC3	FB									
TLR2	IZ									
TLR2	ANL									
TNFRSF21	IZ									
ABI3BP	ANL									
ABI3BP	FB									
CLU	IZ									
COL10A1	IZ									
COL10A1	FB									
COL1A1	IZ									
COL1A1	FB									
GALNT14	IZ									
GALNT14	ANL									
GALNT14	FB									
MGP	IZ									
MGP	FB									
SGMS2	FB									
TIMP2	IZ									

Supplementary Figure 1. Changes in steady state mRNA levels in response to the chondrogenic stimulation relative to the 0 h time point. IZ = interzone cell; ANL = anlagen cell; FB = fibroblast. a) Upregulated genes, b) downregulated genes, and c) genes with mixed patterns of upregulation and downregulation after inducing chondrogenesis. d) Genes with no response to the chondrogenic stimulation.
