# Supplementary material for: Kinetics of Gene Expression Changes in Equine Fetal Interzone and Anlagen Cells Over 14 Days of Induced Chondrogenesis
Source: Front Vet Sci. 2021 Aug 9;8:722324. doi: 10.3389/fvets.2021.722324 (PMC8380811; doi:10.3389/fvets.2021.722324)
Supplement: Supplementary file 6 [file Data_Sheet_2.pdf]

(Time point, h)

Genes	Cell type	Monolayer	0	1.5	3	6	12	24	48	96	168	336
APLNR	IZ	***	Ref.[a]									
APLNR	ANL	***	Ref.									
APLNR	FB	***	Ref.							**	***	***
GDF5	IZ	**	Ref.				***	***	***	***	***	***
GDF5	ANL	*	Ref.			**	***	***	***	***	***	***
GDF5	FB	***	Ref.							**	***	***
S1PR3	IZ	***	Ref.		***	**	*	***	***	***	***	***
S1PR3	ANL		Ref.	**	***	***	**	***	***	***	***	***
S1PR3	FB		Ref.	**	***	***	***	***	***	***	***	***
TLR2	IZ	*	Ref.									
TLR2	ANL	***	Ref.									
TLR2	FB		Ref.								***	***

Upregulated based on 0h	***, P<0.0001
Downregulated based on 0h	**, P<0.01
	*, P<0.05

Supplementary Figure 2. Genes with significantly different steady state mRNA levels between passage 4 monolayer cultures and passage 5 cell pellet cultures at 0 h. IZ = interzone cell; ANL = anlagen cell; FB = fibroblast.

[a]The reference point (baseline) for comparison was 0 h in each gene × cell type combination.
