# Supplementary material for: Kinetics of Gene Expression Changes in Equine Fetal Interzone and Anlagen Cells Over 14 Days of Induced Chondrogenesis
Source: Front Vet Sci. 2021 Aug 9;8:722324. doi: 10.3389/fvets.2021.722324 (PMC8380811; doi:10.3389/fvets.2021.722324)
Supplement: Supplementary file 7 [file Data_Sheet_3.pdf]

a) Genes that were differentially expressed in the indicated pairwise cell type comparison at baseline (0 h) and retained differences after inducing chondrogenesis

Comparison between interzone and anlagen cell cultures

(Time point, h)

Genes	0	1.5	3	6	12	24	48	96	168	336
RUNX3	***	***	***	***	***	***	***	***	***	***
ABI3BP	***	***	***	***	***	***	**	***	**	
MGP	***	***	***	***	***	**				
LEF1	***	***	***	**	***	***	***	***		
BOC	***	***	***	**	**	***	**	***	**	**
SHC3	***	***	**	*		*	**	*		
DLX5	**	***	***	***	***	**				
GDF6	**	**	**	**	***	***	**	***	***	***
RUNX2	**	**			**		*	*		
OMD	**							**	**	
PDLIM1	*								***	***

Comparison between interzone cell and fibroblast cultures

(Time point, h)

Genes	0	1.5	3	6	12	24	48	96	168	336
ASS1	***	***	***	***	***	***	***	***	***	***
LEF1	***	***	***	***	***	***	***	***	***	***
COMP	***	***	***	***	***	***	***	***	***	***
TLR4	***	***	***	***	***	***	***	***	***	*
DLX5	***	***	***	***	***	***	***	***	***	
DCN	***	***	***	***	***	***	***	***	**	***
ENTPD1	***	***	***	***	***	***	**	**	***	**
ABI3BP	***	***	***	***	**	*		*		
SHC3	***	***	**	***	***	***	***	***	***	**
FGF1	***	***	*		***	***	***	***		
AQP1	*	*	**	***	***					
CTGF	*	*	*		*	***	***	***	***	***
RUNX3	*	*		**	**	*	**			
FAM20A	***		***	***	***	**		***	**	**

Comparison between anlagen cell and fibroblast cultures

(Time point, h)

Genes	0	1.5	3	6	12	24	48	96	168	336
ASS1	***	***	***	***	***	***	***	***	***	***
ENTPD1	***	***	***	***	***	***	***	***	***	***
TLR4	***	***	***	***	***	***	***	***	***	***
DLX5	***	***	***	***	***	***	***	***	***	**
DCN	***	***	***	***	**	*				
TNFSF11	***	***	***	***						***
AQP1	***	***	**	***	***	**			**	***
LEF1	***	***	**	**	***					***
FGF1	***	***								
BOC	**	***	**		*	***	***	***	***	*
FAM20A	**	**	**						**	***
IGFBP5	**	**	*							**
ITGA7	**	**		*	*	*				***
DIO2	**	**								**
CDON	**	**								
CTGF	**	*	**	**			**	***	**	
CLU	*	**	*		*	*				**
RUNX3	*							*	***	***

b) Genes that were differentially expressed in the indicated pairwise cell type comparison at baseline (0 h) but lost differences after inducing chondrogenesis

Comparison between interzone and anlagen cell cultures

(Time point, h)

Genes	0	1.5	3	6	12	24	48	96	168	336
DIO2	**									
COL2A1	**									
COMP	**									

Upregulated in interzone cell culture	***, P<0.0001
Upregulated in anlagen cell culture	**, P<0.01
Upregulated in fibroblast culture	*, P<0.05

Annoation group
Trasncription regulation
Signal transduction
Extracellular matrix
Not categorized

c) Genes that were not differentially expressed in the indicated pairwise cell type comparison at baseline (0 h) but responded differently to the chondrogenic stimulation

Comparison between interzone and anlagen cell cultures

(Time point, h)

Genes	0	1.5	3	6	12	24	48	96	168	336
S1PR3		**	**	**						
RELN		*	**	***	***	***	***	***	**	
TNFSF11		**	***	***	**		**	*		
FGF1				**	***	***	**			
SP7				**			*			
BMPR1A				*	*					
SMPD3					***	***	***	***	***	***
FAM20A					***	**		**		
FGF18					**	**		***	***	***
IHH					**			*	***	**
TNFRSF21						**				***
MASP1						*		**	***	***
PRKG2							**	***	***	**
ABCC9							**	**		
SERPINE1							**			
THBS4								***	***	***
FGFR3								***	***	***
S100A1								***	***	***
MET								**	**	***
IBSP								**	*	
COL5A3								**		
PANX3								*		

Annoation group
Trasncription regulation
Signal transduction
Extracellular matrix
Not categorized

Upregulated in interzone cell culture	***, P<0.0001
Upregulated in anlagen cell culture	**, P<0.01
Upregulated in fibroblast culture	*, P<0.05

Comparison between interzone cell and fibroblast cultures

(Time point, h)

Genes	0	1.5	3	6	12	24	48	96	168	336
MGP		**	**	**	**			*		
SNAI1		**								
S1PR3		*	***	***	***			**	**	**
SLC38A1		*								
ADGRG1			**					*	**	
SGMS2			*							
PRKG2				***	***	***	***	***	***	***
GDF5				**	***	***	***	***	*	
MET				**	***	**	*	***	***	***
ALPL					*	**	**			**
FZD1					*	**				
IHH					*			***	**	*
COL5A3					*				**	**
ADAMTS5						***	***	***	***	***
COL2A1						**	***	***	***	***
SERPINE1						**	***	**	***	***
NEFL						**	***			
GLI3						**	**	***	***	***
TNFSF11						*				*
PLAT							**	***	***	***
S100A4							**	**		***
IBSP							*	***	**	
CLU							*			**
ENTPD2							*			
ABCC9							*			
FAM132A								***	***	***
WNT9A								***	***	***
THBS4								***	***	***
TGFBI								***	***	**
MMP2								**	**	*
IGFBP5								**	**	
RELN								*		

Comparison between anlagen cell and fibroblast cultures

(Time point, h)

Genes	0	1.5	3	6	12	24	48	96	168	336
COMP		**	***	***	***	***	***	***	***	***
OSR2		*				*	**			
SLC38A1		*								*
SNAI1		*								
SGMS2			*	*					***	*
GDF5				***	***	***	***	***	***	
IHH				***	***	***	**	***	***	***
COL5A3				**	***	***	***	***	***	***
FZD1				**	***	***	**			
TNFRSF21				**	***	***	*			*
FGF18					***	***	**	**	***	***
SMPD3					**	***	***	***	***	**
RELN					*	***	***	***	***	
ALPL					*	**				***
ADAMTS5						**	**	***	***	***
COL2A1						**	***	***	***	***
SP7						**	*			
MASP1						**		**	***	***
S100A4						*	**	**	***	***
FGFR3								***	***	***
S100A1								***	***	***
WNT9A								***	***	***
PLAT								**	***	***
SERPINE1								**	***	***
SHC3								**	***	
MMP2								**		**
FAM132A								*	***	***
MGP								*		

(continued Supplementary Figure 3.c) Genes that were not differentially expressed in the indicated pairwise cell type comparison at baseline (0 h) but responded differently to the chondrogenic stimulation

Comparison between interzone and anlagen cell cultures

(Time point, h)

Genes	0	1.5	3	6	12	24	48	96	168	336
SNAI2									***	***
CLU									***	***
CDH13									***	***
ENTPD2									***	**
ASS1									**	***
TGFBI									**	***
S100A4									**	***
SGMS2									**	
WNT9A									*	***
SNAI1									*	
ALPK3										***
CREB5										***
ANGPTL4										***
AQP1										***
TSPAN15										***
COL1A1										***
TIMP2										***
CTGF										**
PLVAP										**
ITGA7										**
GLI3										*
SLC38A1										*
ADAMTS5										*
TLR2										*
TLR4										*

Comparison between interzone cell and fibroblast cultures

(Time point, h)

Genes	0	1.5	3	6	12	24	48	96	168	336
GALNT14									***	
FGFR3									**	***
RUNX2									**	**
FRZB									*	*
IGFBP7										***
ANGPTL4										**
APLNR										**
FGF18										*
TLR2										*

Comparison between anlagen cell and fibroblast cultures

(Time point, h)

Genes	0	1.5	3	6	12	24	48	96	168	336
ENTPD2									***	***
PDLIM1									***	***
SNAI2									***	***
ANGPTL4									***	***
CDH13									***	***
GDF6									***	***
PANX3									***	*
APLNR									**	***
COL1A1									**	***
BMPR1A									**	
ABI3BP									**	
ADGRG1									*	**
CREB5										***
NTRK2										***
TLR2										***
IGFBP7										**
ITGAV										**
TSPAN15										**
TIMP2										**

Upregulated in interzone cell culture	***, P<0.0001
Upregulated in anlagen cell culture	**, P<0.01
Upregulated in fibroblast culture	*, P<0.05

Annoation group
Trasncription regulation
Signal transduction
Extracellular matrix
Not categorized

d) Genes that were not differentially expressed in the indicated pairwise cell type comparison at baseline (0 h) and after inducing chondrogenesis

Comparison between interzone and anlagen cell cultures

Genes
ENTPD1
OSR2
ADGRG1
APLNR
FAM132A
GDF5
IGFBP5
IGFBP7
PLAT
ADGRG2
BMP2
CDON
FRZB
FZD1
ITGAV
KCNJ8
LOC100630171
NTRK2
PTCH2
ALPL
DCN
MMP2
SPARCL1
COL10A1
GALNT14
NEFL

Comparison between interzone cell and fibroblast cultures

Genes
ALPK3
CREB5
OSR2
PDLIM1
SNAI2
SP7
ADGRG2
BMP2
BMPR1A
BOC
CDH13
CDON
DIO2
GDF6
ITGAV
KCNJ8
LOC100630171
NTRK2
PANX3
PLVAP
PTCH2
S100A1
TNFRSF21
TSPAN15
COL10A1
COL1A1
ITGA7
OMD
SMPD3
SPARCL1
TIMP2
MASP1

Comparison between anlagen cell and fibroblast cultures

Genes
ALPK3
GLI3
RUNX2
ABCC9
ADGRG2
BMP2
FRZB
IBSP
KCNJ8
LOC100630171
MET
PLVAP
PRKG2
PTCH2
S1PR3
COL10A1
GALNT14
OMD
SPARCL1
THBS4
TGFBI
NEFL

Annoation group
Trascnription regulation
Signal transduction
Extracellular matrix
Not categorized

Supplementary Figure 3. Four patterns of differential gene expression before and after inducing *in vitro* chondrogenesis. Each pattern is shown in a), b), c), and d), respectively.
