# Supplementary material for: Kinetics of Gene Expression Changes in Equine Fetal Interzone and Anlagen Cells Over 14 Days of Induced Chondrogenesis
Source: Front Vet Sci. 2021 Aug 9;8:722324. doi: 10.3389/fvets.2021.722324 (PMC8380811; doi:10.3389/fvets.2021.722324)
Supplement: Supplementary file 8 [file Data_Sheet_4.pdf]

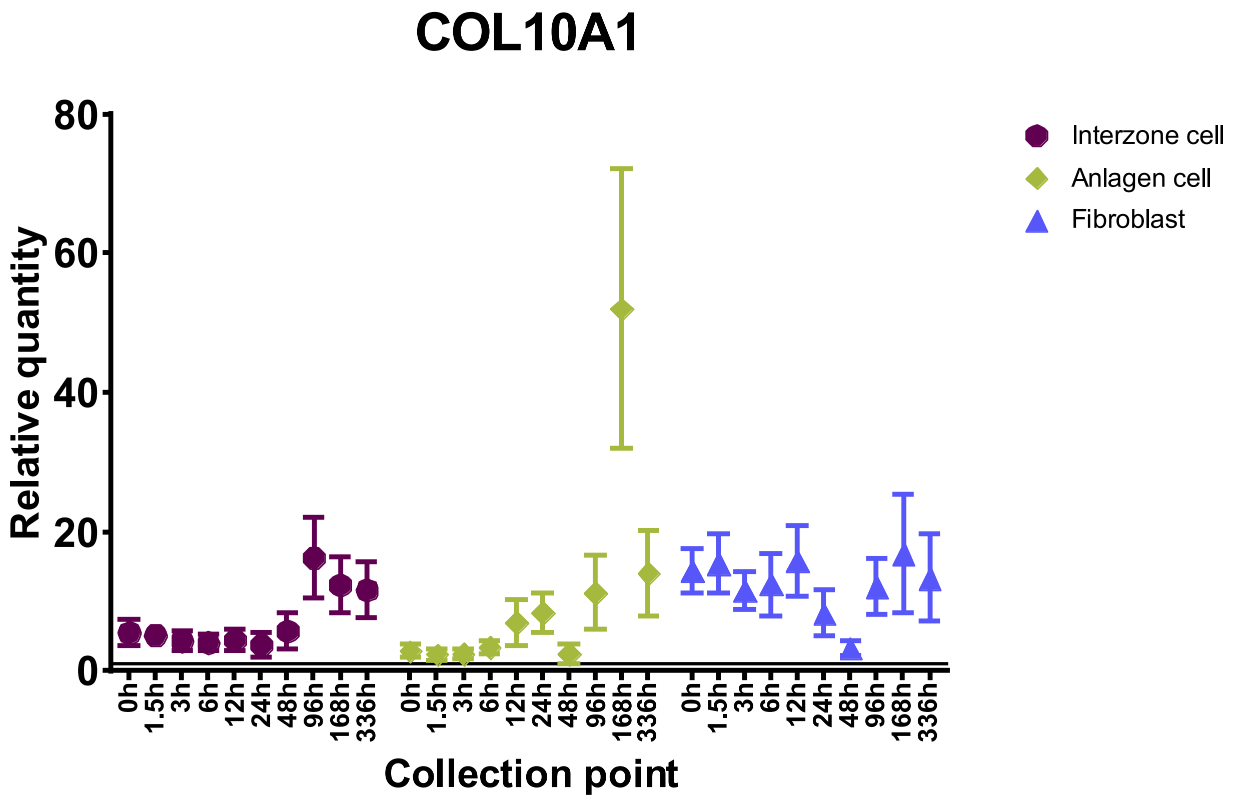

Supplementary Figure 4. Steady state mRNA levels of COL10A1. Relative quantities were calculated based on the positive control sample (pooled equine adult tissue and fetus RNA). Mean ± SEM (n=7).
